# Kinetic Study of the Effective Thermal Polymerization of a Prebiotic Monomer: Aminomalononitrile

**DOI:** 10.3390/polym15030486

**Published:** 2023-01-17

**Authors:** Carlos Hortelano, Marta Ruiz-Bermejo, José L. de la Fuente

**Affiliations:** 1 Instituto Nacional de Técnica Aeroespacial “Esteban Terradas” (INTA), Ctra. de Torrejón-Ajalvir, km 4, Torrejón de Ardoz, 28850 Madrid, Spain; 2 Centro de Astrobiología (CAB), CSIC-INTA, Ctra. de Torrejón-Ajalvir, km 4, Torrejón de Ardoz, 28850 Madrid, Spain

**Keywords:** HCN polymers, aminomalononitrile, bulk polymerization, DSC, kinetic, mechanism

## Abstract

Aminomalononitrile (AMN), the HCN formal trimer, is a molecule of interest in prebiotic chemistry, in fine organic synthesis, and, currently, in materials science, mainly for bio-applications. Herein, differential scanning calorimetry (DSC) measurements by means of non-isothermal experiments of the stable AMN *p*-toluenesulfonate salt (AMNS) showed successful bulk AMN polymerization. The results indicated that this thermally stimulated polymerization is initiated at relatively low temperatures, and an autocatalytic kinetic model can be used to appropriately describe, determining the kinetic triplet, including the activation energy, the pre-exponential factor, and the mechanism function (*E_α_*, *A* and *f*(*α*)). A preliminary structural characterization, by means of Fourier transform infrared (FTIR) spectroscopy, supported the effective generation of HCN-derived polymers prepared from AMNS. This study demonstrated the autocatalytic, highly efficient, and straightforward character of AMN polymerization, and to the best of our knowledge, it describes, for the first time, a systematic and extended kinetic analysis for gaining mechanistic insights into this process. The latter was accomplished through the help of simultaneous thermogravimetry (TG)-DSC and the in situ mass spectrometry (MS) technique for investigating the gas products generated during these polymerizations. These analyses revealed that dehydrocyanation and deamination processes must be important elimination reactions involved in the complex AMN polymerization mechanism.

## 1. Introduction

Aminomalononitrile (AMN), as the formal trimer of hydrogen cyanide (HCN), has been largely considered in prebiotic chemistry studies as a precursor of *N*-heterocycles such as purine and pyrimidine nucleobases [1,2,3] or pteridines [4], and also for the plausible abiotic formation of peptide-like compounds [5]. Inspired by this chemistry, recently, the use of AMN in multicomponent syntheses for the production of peptide nucleic acid analogs [6] and amino imidazole carbonitriles with antivirus activity [7] has been described. Moreover, its spontaneous polymerization under slight alkaline aqueous conditions to generate AMN-based films with potential biomedical applications has been described—for example, as coatings in bone-contacting medical devices [8] and protective coatings against corrosion [9]. Following this growing interest in the AMN in the development of a new family of multifunctional materials, loose nanofiltration membrane surface functionalization for achieving efficient molecular separation [10] and biocompatible and multifunctional coatings with antifouling, antibacterial, and self-healing properties have been described [11]. Significantly, AMN is a yellow oil that evolves quickly into a dark-brown tarry mass [3,5]. Therefore, it is generally stabilized as its tosylate salt [12], and in this way, it is commercialized as aminomalononitrile *p*-toluenesulfonate salt (AMNS). Consequently, all the syntheses and aqueous polymerizations indicated above correspond with the use of AMNS as the main reactant.

Another one of the oligomers of the HCN—in this case, its formal tetramer, the diaminomaleonitrile (DAMN)—has been recently successfully thermally polymerized, both in an aqueous medium and in bulk, and its polymeric systems have demonstrated interesting electrochemical properties and applications [13,14,15]. The highly efficient polymerization of DAMN, even in a solid-state, offers alternative approaches to environmentally favorable processes with a relatively low cost. It is important to take into account that these macromolecular systems, derived from the HCN and inspired by prebiotic chemistry, provide wide design opportunities for the production of a new generation of multifunctional materials due to the ease of their syntheses and the possibility of tailoring their material properties by tuning the reaction conditions applied and choosing different monomers [16,17]. In addition, they present a great analogy with graphitic carbon nitrides, which belong to the family of two-dimensional conjugated polymers with carbon and nitrogen in the polymer backbone. These semiconducting materials have gained considerable attention among researchers across the world due to their excellent physical and chemical properties [18].

Therefore, taking into account the promising applications of the HCN-derived polymers, their resemblance with the well-known carbon nitrides, the free-solvent highly efficient thermal polymerization of DAMN for the generation of redox electroactive systems, and the great interest in AMN as a synthon in prebiotic and organic syntheses and as monomer in the design of polymeric coatings, the aim of this study is to explore the thermal stability of AMNS by means of differential scanning calorimetry (DSC) measurements. This technique, in addition to simultaneous thermal analysis coupled with mass spectrometry (TG-MS), was chosen to analyze AMNS under dynamic thermal conditions, since they have been proven to be extremely useful in understanding the kinetic, thermodynamic, and likely pathways of DAMN polymerization. TG-MS has allowed for the evaluation of gaseous species generated during the polymerization reactions to gather more information about the mechanism [19,20]. This fact has motivated the choice to apply this methodology to the thermal reaction of AMNS to know the relevant aspects of such process. Moreover, microstructural characterization by Fourier transform infrared (FTIR) spectroscopy was also performed to identify the final products after the thermal treatments of the AMNS, demonstrating the effective and rapid polymerization of AMN, resembling the macromolecular systems from the thermal DAMN polymerization. Thus, the main goals of this work are to obtain a more comprehensive understanding of the thermal stability of AMNS and the nature of the final polymeric products obtained, taking into account that they are deserving of further research in advanced multifunctional polymer materials science and technology, and to establish a hypothetical mechanism of formation for these polymeric structures.

## 2. Materials and Methods

### 2.1. Materials and Thermokinetic Analysis

AMNS (98%) was purchased from Sigma Aldrich (m.p. 174 °C) and used as received. DSC measurements were performed on a DSC 7 (Perkin Elmer, Norwalk, CT, USA) calibrated with high-purity chemicals. Temperature-ramping DSC studies were performed from 25 to 250 °C, in accordance with the previous studies on the thermal polymerization of DAMN [19,20], at heating rates (*β*s) of 2.5, 5, 10, 15, 20, and 25 °C/min in a dry nitrogen atmosphere at a rate of 20 mL/min, using approximately 4–5 mg of AMNS placed in aluminium pans. After the non-isothermal reactions, the samples were rapidly cooled to room temperature, and heating scans at 20 °C/min were performed. Each test was repeated at least two times to ensure consistent results, showing a high reproducibility. The reaction heats were estimated by connecting the baseline before and after the peak with a straight line and integrating the area under the peak. The final values are the average of several measurements taken for each sample. Additional DSC measurements were carried out at *β* = 10 °C/min under an air atmosphere and also using sealed pans in order to examine how these experimental variables affect the thermal process under study. Thermogravimetry (TG), differential scanning calorimetry (DSC), and differential thermal analysis (DTA) measurements were performed using a simultaneous thermal analyzer model (SDTQ-600/Thermo Star) of TA Instrument^®^ (New Castle, DE, USA). The non-isothermal experiments were carried out under dynamic conditions from room temperature to 250 °C at a *β* of 10 °C/min under an argon atmosphere. The average sample weight was ~10 mg, and the argon flow rate was 100 mL/min. A coupled TG-mass spectrometer (TG-MS) system equipped with an electron-impact quadrupole mass-selective detector (model Thermostar QMS200 M3 of TA Instrument^®^) was employed to analyze the main species that evolved during the dynamic thermal analysis of the AMNS samples. The rest of the *p*-toluenesulfonic acid (HOTS) from the black formed polymeric products after the thermal treatment was eliminated after successive washing with water for the FTIR characterization of the samples. Conventional TG analyses from 25 to 900 °C at 10 °C/min and under nitrogen flow using a TGA 7 (Perkin Elmer, Norwalk, CT, USA) were also carried out to complete the characterization of the final products after thermal treatment.

The DSC fractional conversion *α* was calculated using the following equation:

(1) $$\alpha =\frac{\int_{0}^{t}(dH/dt)dt}{\Delta H_{total}}$$

where d*H*/d*t* is the DSC heat flow, *t* is the reaction time, and Δ*H_total_* is the total heat evolved. The reaction rate (d*α*/d*t*) can be derived from Equation (1).

(2) $$\frac{d\alpha }{dt}=\frac{dH/dt}{\Delta H_{total}}=k\left(T\right)f\left(\alpha \right)$$

where the rate constant *k*(*T*) is a temperature-dependent reaction rate constant expressed by the Arrhenius equation, and *f*(*α*) is the kinetic model function representing the underlying polymerization mechanism. From Equation (2), and considering that d*α*/d*t* = *β* (d*α*/d*T*), it is possible to obtain:

(3) $$\beta \frac{d\alpha }{dT}=Aexp\left(\frac{-E_{a}}{RT}\right)f\left(\alpha \right)$$

where *T* is the temperature, *A* is the pre-exponential factor, *E_a_* is the apparent activation energy, and *R* is the gas constant. Due to its simplicity, the most popular approach to determining *E_a_* is the Kissinger method [21], where *E_a_* is evaluated through the peak exothermic absolute temperatures (*T_p_*) at a maximum rate. Considering that this method is only valid for a one-step process, it follows the form:

(4) $$\ln\left(\frac{\beta }{T_{p}^{2}}\right)=\ln\left(\frac{AR}{E_{a}}\right)-\frac{E_{a}}{RT_{p}}$$

Alternatively, *E_a_* can be calculated through iso-conversional methods. Different integral methods, including the Ozawa–Flynn–Wall (OFW) [22], Kissinger–Akahira–Sunose (KAS) [23], and Starink [24] methods, can be used. Among them, the latter can achieve more precise values of *E_α_*, offering a general formula with the following form:

(5) $$\ln\frac{\beta_{i}}{T_{\alpha ,i}^{B}}=constant-C\left(\frac{E_{\alpha }}{RT_{\alpha ,i}}\right)$$

where *B* and *C* are the parameters determined by the type of temperature integral approximation, and *T_α,i_* is the temperature at the *β_i_* for a conversion *α*. Therefore, (*B*, *C*) = (0, 1.052) under the OFW method, (*B*, *C*) = (2, 1) under the KAS method, and (*B*, *C*) = (1.92, 1.0008) under the Starink approach. The accuracy of these methods depends on the numerical approximation obtained by solving the temperature-dependent integral term of the Arrhenius constant. Another way to obtain the *E_α_* values is by means of a differential iso-conversional method, such as the one proposed by Friedman based on the following equation [25]:

(6) $$\ln\frac{d\alpha }{dt}=\ln\left[Af\left(\alpha \right)\right]-\frac{E_{\alpha }}{RT}$$

Although there are several models for describing the non-isothermal polymerization reaction kinetics, the Málek statistical analysis method is often adopted to derive the kinetic equation [26]. The most suitable kinetic model can be determined by the functions *y*(*α*) and *z*(*α*), which are established from the experimental data as follows:

(7) $$y\left(\alpha \right)=\left(\frac{d\alpha }{dt}\right)e^{x}$$

(8) $$z\left(\alpha \right)=\pi \left(x\right)\left(\frac{d\alpha }{dt}\right)\frac{T}{\beta }$$

where *x* represents the reduced activation energy (*E_α_*/*RT*), and *π*(*x*) is the expression of the temperature-integral, which can be approximated well with Equation (9) [27]:

(9) $$\pi \left(x\right)=\frac{x^{3}+18\;x^{2}+88\;x+96}{x^{4}+20\;x^{3}+120\;x^{2}+240\;x+120}$$

According to the Málek method, *E_α_* should be determined independently through other methods, after which the function curves of *y*(*α*) and *z*(*α*) can be obtained. From both curves, the conversion *α_M_* at the maximum value of *y*(*α*) and the conversion *α_p_^∞^* at the maximum value of *z*(*α*) can be obtained. By comparing these parameters and determining whether they meet a specific relationship, a suitable kinetic model can be identified [26].

### 2.2. FTIR Spectroscopy

The polymeric samples obtained after DSC analyses were further evaluated by Fourier transform infrared (FTIR) spectroscopy. The spectra were acquired in the 4000–400 cm^−1^ spectral region using an FTIR spectrometer (Nicolet^®^, model NEXUS 670, Pleasantville, NY, USA) configured with a drift-reflectance accessory (Harrick, model Praying Mantis DRP, Gaithersburg, MD, USA) mounted inside the instrument compartment. The spectra were obtained in CsI pellets, and the spectral resolution was 2 cm^−1^.

## 3. Results and Discussion

### 3.1. Dynamic Thermal Analysis of AMNS and FTIR Study

**Figure 1:**
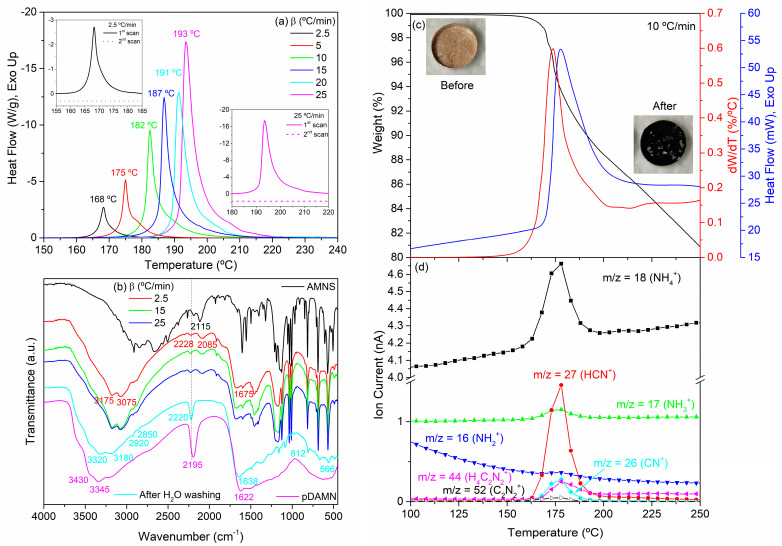
(**a**) DSC curves of the AMNS at different heating scans; (**b**) FTIR spectra of the AMNS, of the reaction product obtained under dynamic DSC conditions and nitrogen atmosphere, and after H_2_O washing, as well as of HCN polymers obtained from DAMN; (**c**) TG, DTG, and DSC curves and (**d**) temperature-dependent ionic current variation of the AMNS polymerization at 10 °C/min under argon atmosphere.

As a first approach to determining the thermal stability of the AMNS and the likely thermal polymerization of AMN, in a similar way to that undergone by the DAMN, a primary DSC study was carried out. Figure 1a shows the DSC thermograms of AMNS at different *β*s between 2.5 and 25 °C/min under nitrogen atmosphere, and the corresponding parameters obtained from these curves are listed in Table 1. As shown in the thermograms, there is a very similar behavior of the experiments performed at distinct rates, with a narrow sharp exothermic peak in the range of 170–195 °C and an evolved heat (Δ*H*) close to 300 J/g. The values for the initial temperature (*T_i_*), peak temperature (*T_p_*), and final temperature (*T_f_*) are shown in Table 1. From these first results, no endothermic peaks related with the melt of the AMNS are observed at any of the *β*s. This result questions the correct identification of the melting temperature of the AMNS pointed out in the literature (175–176 °C [3], 168–169 °C [12], and 169–171 °C [28]) and in the specifications of the commercial AMNS. Therefore, the unique exothermic process close to ~175 °C might be related with a very rapid exothermic decomposition, which masks the melting endothermic process in an analogous way to that suffered by the 2-oximemalononitrile [29] or with a polymerization process similar to the thermal polymerization of DAMN. For this compound, to lower the temperature, before the melting point, the peak was detected due to the solid-state polymerization of DAMN and, at a higher temperature, its melting polymerization [19,20].

At this point, a detailed FTIR analysis was completed in order to determine if the AMNS undergoes decomposition or polymerization after a thermal treatment. Figure 1b shows the IR spectrum of the commercial AMNS together with some of the samples obtained after the dynamic heating. The spectral features of both samples are different, although they share certain narrow and well-defined peaks at low wavenumbers—for example, 1033, 1008, 812, 688, and 566 cm^−1^, which can be assigned to the HOTS and remain observable and invariant during the thermal transformation, i.e., the HOTS is not altered during the heating. However, a significant change was found for the X–H region (N–H and C–H); thus, for AMNS, the stronger peaks appears at 2912 and 2670 cm^−1^ in the C–H range, and after the thermal dynamic scans, two broad peaks are now located at 3175 and 3075 cm^−1^ in the N–H range. In the nitrile region, the most intense peak at 2115 cm^−1^ for the AMNS is now apparently diminished and shifted to 2228 cm^−1^. However, a strong absorption in the double bond range, due to C=C and/or C=N (1675 cm^−1^), is developed during the exothermic alteration. Thus, according to this figure, nitrile moieties are transformed into other functionalities, with an intensity change in both the double bond and amine ranges, and, then, this thermochemical reaction could be related to a polymerization process.

After a water washing procedure for these samples, the FTIR spectra again changed significantly due to the practical lack of the bands related to the HOTS. Now, these spectra (light-blue line in Figure 1b, as a representative example) show broad peaks in all of the spectral regions and present some resemblance to those reported by Evans and co-workers, who studied the polymeric coatings obtained from aqueous AMN polymerizations [30]. Moreover, Figure 1b contains a spectrum of the polymers obtained from DAMN also by means of the dynamic thermal treatment, and a high structural similarity between the AMNS heating and DAMN thermal polymerization is observed. Therefore, without a doubt, the dynamic heating of the AMNS leads to the polymerization of the AMN after the release of HOTS. Furthermore, no melt could be observed before polymerization, in agreement with the fact that the pure AMN is an oil [3,5]. Thus, this result indicates that both nitrile monomers, AMN and DAMN, can be thermally polymerized, yielding a conjugated heterocyclic system with isolated or conjugated nitrile groups, C≡N [19,20]. In the present particular case, the broad and poorly resolved region centred at ~1640 cm^−1^ could correspond to heterocyclic systems, since, in general, azoles have three or four bands in the region of 1670–1320 cm^−1^ due to C=C and C=N stretching, with intensities that depend on the substituent, the position, and the nature of the ring heteroatoms [31]. Polymers containing imidazole rings are expected considering the Thissen and Evans studies based on the AMN [8,30]; for example, imidazoles present several bands of variable intensities in the range of 1660–1450 cm^−1^ due to the mentioned system [31]. Therefore, all the spectroscopic data shown in Figure 1b indicate that the raw end product obtained after the thermal process applied to AMNS is a conjugated polyheterocyclic system plus HOTS.

Once the thermal polymerization of AMNS has been demonstrated, additional aspects must be considered from Figure 1a and the corresponding data collected in Table 1. Thus, it is observed that, as *β* increases, the exothermic peak is shifted to a higher temperature, increasing the peak maximum from 168 to 193 °C, with an increase in the rate from 2.5 to 25 °C/min. This shift in the DSC peaks is accompanied by a variation in intensity, which is reflected in the maximum values of the polymerization rate, as is described later. The same figure also shows the asymmetric profile of the DSC curves obtained at higher *β*s, this feature being especially relevant at 5 °C/min, where a shoulder in the high-temperature range can be seen. This result indicates that the thermal polymerization of AMNS could be a complex process, as is discussed later. In addition, the graph inset shows the second heating scans of the samples polymerized at 2.5 and 25 °C/min. These DSC thermograms confirmed that no further thermal reactions occurred after the nonisothermal polymerizations used here, which resulted in a practically complete, attainable conversion. However, we take into account that a slight decrease in the estimated value of the heat of the polymerization is observed when the heating runs increase, as is displayed in Table 1. It is important to note that, at the highest *β*s, the reaction is extremely fast, with polymerization times shorter than 3 min. A polymerization enthalpy of 307 J/g for the AMNS should be taken as a reference for the subsequent kinetic assessment, as it is the highest value found when the *β* is 2.5 °C/min. In any case, this reaction heat is weaker than that described for the bulk thermal polymerization of DAMN, where values of 520 and 810 J/g were found for its solid-state polymerization and in the melt, respectively [19,20]. In the present study, we do not start with the free monomer as an initial reagent but with a salt, and, therefore, the corresponding process that releases AMN must be considered, which might be endothermic, decreasing the global exothermicity of the reaction under study.

On the other hand, since the FTIR spectra from the thermal polymerization of AMNS and DAMN (Figure 1b) are very similar, the pathways of production for these *N*-heterocyclic macrostructures may be considered. Mechanistic studies of the bulk thermal polymerization of DAMN have revealed that different elimination reactions, such as dehydrocyanation and deamination, take place during the course of these reactions according to TG-MS measurements [19,20]. Consequently, the use of the TG-DSC-MS technique with an equivalent temperature program will help in testing if these elimination reactions that accompany the DAMN polymerization also take place during the AMNS polymerization.

### 3.2. Simultaneous TG-DSC-MS Analysis

Figure 1c shows the TG curves obtained at a *β* of 10 °C/min under an inert atmosphere of argon. Under these conditions, a mass loss of about 20% from room temperature to 250 °C was observed, which is the same temperature range as that of the DSC measurements discussed above. A similar weight loss was detected for DAMN when analogous thermal stress was applied [19,20]. This suggests that both AMNS and DAMN undergo a thermal polymerization, where certain elimination reactions must be contemplated. In addition, while for both the solid-state and melt polymerization of DAMN, this mass loss occurs until a constant weight is obtained (Figures 8 and 9 from the reference [20] and Figure 7a from the reference [19]), in the present case, considering the thermal interval shown in Figure 1c, a continuous and unfinished weight loss is observed for AMNS. These results seem to indicate different elimination mechanisms during the polymerization thermal processes for the two monomers derived from HCN, as is discussed below. On the other hand, the shape of the derivative thermogravimetric (DTG) curve is very similar to that extracted from the simultaneous DSC trace, as is shown and discussed in more detail in Figure 1a.

These results encourage the application of a coupled MS technique to obtain insights into the thermal polymerization mechanism of AMNS through the elucidation of the volatile species generated. The MS curves of the main gases produced in the thermochemical reactions detected during this study are plotted in Figure 1d. The presence of the most intense ion fragment of *m*/*z* = 18 (NH_4_^+^), 27 (HCN^+^), 16 (NH_2_^+^), 17 (NH_3_^+^) and 26 (CN^+^) were attributed to the deamination and dehydrocyanation reactions during this thermal process. The other lower-intensity MS signals with *m*/*z* = 44 can be recognized as formamidine ^+^HN=CH-NH_2_; the fragmented ion observed at *m*/*z* = 52 is proposed to eliminate ^+^CH=CH-CN, which is similar to the ions with *m*/*z* = 26 (^+^CN) and 27 (^+^HCN). These ionic current variations with the increase in the temperature for these fragments are consistent with the loss mass rate curve (DTG), which presents a similar profile in the range between 150 and 200 °C. Interestingly, for DAMN thermal polymerization, especially in melt, a simultaneous release of HCN and NH_3_ was observed according to their TG-MS curves, with the intensity of the ion current corresponding with the *m*/*z* = 27 and 17 fragments [19]. However, a higher loss of NH_4_^+^ against HCN or NH_3_ is observed in the present case, with a clearly lower ion current intensity for the fragment *m*/*z* = 17. The evolution of HCN is in agreement with the pioneering observation made by Matthews in his work about the plausible prebiotic chemistry of the AMN [5]. Therefore, although both AMN and DAMN undergo eliminations such as dehydrocyanation and deamination during their respective thermal polymerizations, it seems that these reactions take place through different processes due to the differences found in the methods of the loss of ammonium and hydrogen cyanide.

Finally, note that no *m*/*z* fragments related to the HOTS were observed, indicating the high stability of this compound after the thermal conditions used. These results are in agreement with the data obtained from FTIR spectroscopy, showing the apparent inaction of this acid during the AMN thermal polymerization.

### 3.3. Kinetic Modelling of AMNS Polymerization

**Figure 2:**
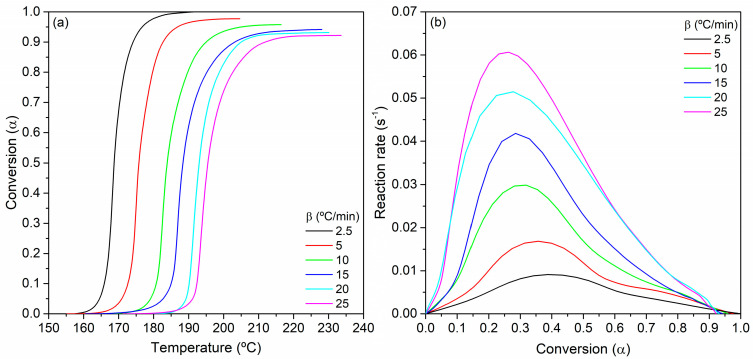
(**a**) Conversion degree *α* versus temperature of the AMNS polymerization under a dynamic DSC mode and nitrogen atmosphere; (**b**) conversion rate d*α/*d*t* versus conversion degree *α*.

Kinetic parameters, such as the reaction order, rate constant, and activation energy, were calculated from the non-isothermal DSC thermograms using the methodology described in Section 2.1. The reaction model of the thermal polymerizations of AMNS can be determined from the kinetic curve type, as shown in Figure 2a, which shows the variation in DSC conversion *α* as a function of temperature at *β*s from 2.5 to 25 °C/min. All these curves show a typical sigmoidal shape, indicating that the system under study followed an autocatalytic mechanism to some extent. As the geometric shape and the distance between curves are almost the same, it is possible to suggest that the different *β*s resulted in comparable kinetics without affecting the fundamental reaction mechanism.

To further analyze AMNS bulk polymerizations, the corresponding reaction rate profiles d*α*/d*t* were plotted as a function of conversion (Figure 2b). The parabolic shape of the polymerization rate indicates that maximum values, *α_p_*, occurred at a certain intermediate conversion, confirming the autocatalytic nature of the reaction, whose values are listed in Table 2. The conversions at the maximum reaction rate oscillate between a value of 0.40 for the lowest heating scan of 2.5 °C/min and a *α_p_* ≈ 0.28 when the *β* increases. All the polymerization rates have similar shapes and peaks, but with dissimilar orders of magnitude, decreasing when the *β* does. In comparison, note that the polymerization of DAMN is a typical single-step reaction only when it occurs under dynamic thermal conditions in melt [19], since a more complex process takes place during the DAMN polymerization in a solid-state under analogous dynamic conditions, with three stages properly identified [20]. The AMNS polymerization could present a similar or even greater complexity than DAMN, since AMN has to be produced from the salt, and, therefore, its thermally induced polymerization takes place in the presence of HOTS. However, the overall process, as shown in Figure 1a, can be assimilated to a single-step process for the sake of simplicity and the comparison with the kinetic parameters of DAMN, and taking into account that AMN cannot polymerize in a solid-state in any case since it is an oil, as was discussed above.

With the goal of determining the kinetic triplet (*E_a_*, *A*, *f(α)*), the apparent *E_a_* is one of the most important parameters. The Kissinger method is one of the simplest methods (Equation (4)) since it can be used independently of the transformation process—in this case, a thermal polymerization. Despite this, it assumes a single-step mechanism, as was mentioned previously, and this method provides an initial approximation of this relevant kinetic parameter. From *T_p_* values, the corresponding Kissinger plot was carried out, and after the linear fitting, an *E_a_* of 147.9 ± 3.6 kJ/mol was calculated. This value is in the range of those data for other prebiotic monomers, such as the DAMN, for which 190.4 and 95.96 kJ/mol have been found for its solid-state and melt polymerizations, respectively, and both *E_a_*s estimated by the same traditional method [20].

**Figure 3:**
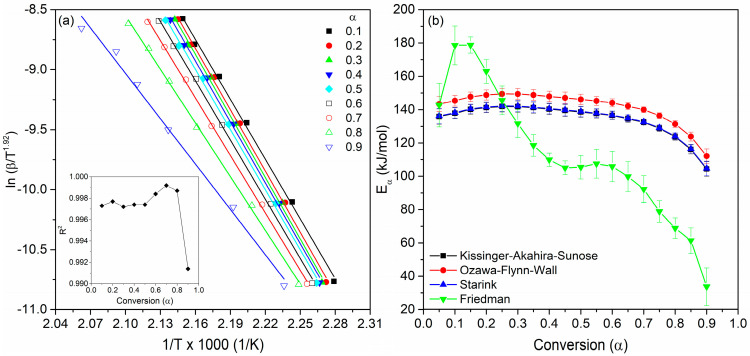
(**a**) Plot corresponding to the Starink method; (**b**) variation in activation energy with the conversion of the AMNS polymerization under a dynamic DSC mode and nitrogen atmosphere.

Indeed, it is well known that iso-conversional methods can be used for exploring the mechanism of polymerization processes through the analysis of the variation in the effective activation energy with the conversion degree [32,33]. From Equation (5), a plot of ln (*β_i_*/*T_αi_*^1.92^) as a function of the 1/*T_α_* value at the same fractional extension of conversion for *α* = 0.05–0.90 is shown in Figure 3a, in accordance with the Starink method. A good linear relationship is observed, as can be observed through the values of the regression coefficients obtained, which are represented in the plot inset. Therefore, the *E_α_*-*α* dependency is shown in Figure 3b, where the values used in this plot were determined by the three integral methods and the Friedman approach, which were described in a previous section. The *E_α_* values indicate few differences between the integral methods used. There is a decrease in *E_α_* from 140 to 105 kJ/mol with the increasing conversion, though the variation in *E_α_* with the extension of the reaction in a range of *α* = 5–70% is insignificant, which indicates that the process appears to be single-step kinetics [34]. This characteristic decrease in the *E_α_* values at the later stages of the reaction has been identified during the bulk DAMN melt polymerization [19], as well as in the production of classical and well-known highly crosslinked polymers—for example, epoxy resins [33]. The lower values of *E_α_* are consistent with those for the diffusion of small molecules in a liquid–solid medium, and the effect was explained by the diffusion control that is associated with vitrification. This effect must be confirmed in future studies. The same behavior was found when the most popular differential method proposed by Friedman was used. Although this method is non-approximative, it is sensitive to data noise, which results in numerical instability [32], and a higher variation in this important kinetic parameter is obtained, as is clearly observed in this figure.

**Figure 4:**
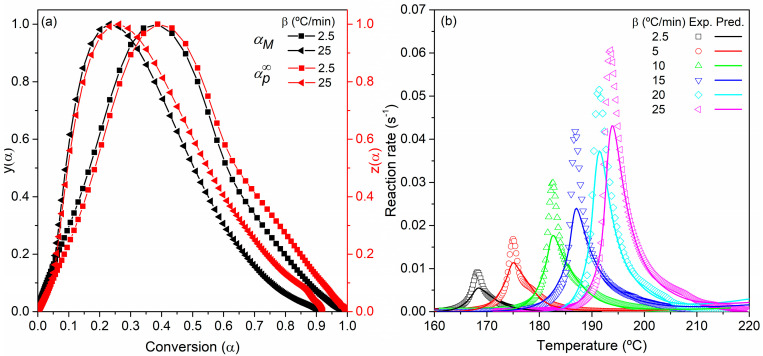
(**a**) Normalized functional curves of *y*(*α*) and *z*(*α*); (**b**) comparison of the experimental reaction rate d*α/*d*t* and that predicted from the SB model for the AMNS polymerization under a dynamic DSC mode and nitrogen atmosphere. Points: experimental values of the conversion rate. Solid lines: calculated values.

According to the Málek methodology, the normalized function curves of *y*(*α*) and *z*(*α*) were constructed according to Equations (7) and (8) (Figure 4a, Table 2). The peak values of *y*(*α*) appeared at a conversion *α_M_* of 0.26–0.40, while in the *z*(*α*) curves, the peak values (*α_p_^∞^*) showed slightly higher conversions. A closer examination of these kinetic parameters indicates that they satisfy the following conditions: 0 < *α_M_* < *α_p_^∞^*, and *α_p_^∞^* ≠ 0.632, which is a strong indication that the experimental data can fit the phenomenological autocatalytic model given by the Šesták–Berggren (SB) model or the expanded Prout–Tompkins model, *f*(*α)* = *α^m^* (1−*α*)*^n^*, where *m* and *n* are reaction orders [35]. Replacing the function in Equation (3), the following expression is obtained:

(10) $$\frac{d\alpha }{dt}=Aexp\left(\frac{-E_{a}}{RT}\right)\alpha^{m}{\left(1-\alpha \right)}^{n}$$

From this equation, linear iterations have been described to obtain the kinetic parameters *m* and *n*, as was the case in the study of the dynamic polymerization of DAMN [19]. However, in this case, a multivariate nonlinear regression method was used to calculate the reaction orders and the frequency factor *A*, followed by applying natural logarithms:

(11) $$\ln\left[\left(\frac{d\alpha }{dt}\right)exp\left(\frac{E_{\alpha }}{RT}\right)\right]=\ln A+m\;\ln\alpha +n\;\ln\left(1-\alpha \right)$$

The values of *m*, *n*, and ln *A* were obtained with acceptable accuracy, with the square of the correlation coefficients *R^2^* ≥ 0.960, finding the highest correlation for the polymerizations at 2.5 °C/min, with an *R^2^* = 0.993. A weak variation in both the reaction order *m* ≈ 1 and the pre-exponential factor is found when the *β* increases. However, an increase in *n* is found, from a value of 1.5 at 2.5 °C/min to a practically doubled value at the highest *β* under study (Table 2). On the other hand, these kinetic parameters can be compared with those obtained for the dynamic melt polymerization of DAMN, finding a high concordance with the reaction orders but a lower frequency factor than that estimated here [19].

To validate the predictability of the obtained kinetic parameters, the corresponding rate curves at the different *β*s were constructed, as shown in Figure 4b. The predicted data were consistent with the experimental rate, even though the calculated curves obtained from the SB model deviate from the experimental points at conversions where the highest rate of polymerization occurs, as is illustrated in this figure. All these results lead to the conclusion that the SB model, assuming termination reactions, normally expressed as *f(α)* = *α^m^* (1−*α*)*^n^* (−ln(1−*α*))*^p^*, could be considered, and the use of a multi-term SB autocatalytic kinetic model could also be raised. This methodology has been necessary when analyzing the DAMN polymerization under isothermal conditions [36]. Therefore, new polymerization reactions under isothermal conditions will be planned in a future work in order to verify the preliminary kinetic analysis presented here. Isothermal measurements are generally better at distinguishing different polymerization mechanisms, and they are essential and mandatary in ensuring a reliable model-guided design at larger scales [37,38], given the high potential of the new polymeric materials developed here.

### 3.4. Mechanistic Considerations

**Figure 5:**
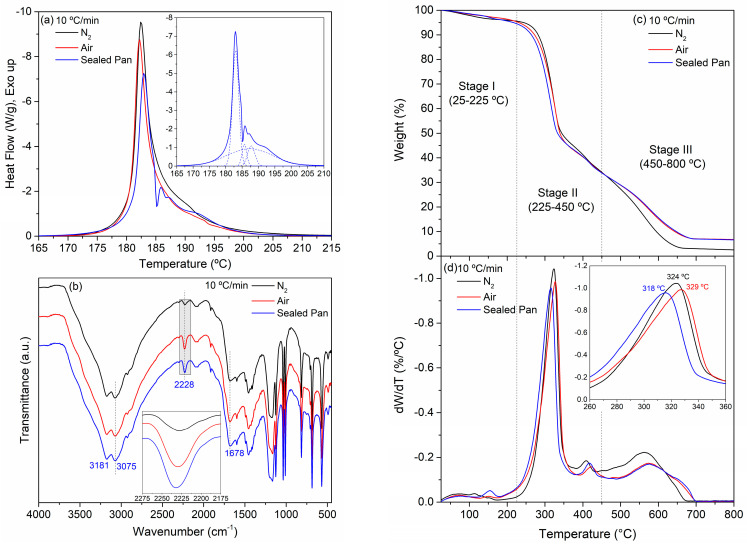
(**a**) DSC thermograms of the AMNS polymerization by means of dynamic DSC under nitrogen and air and using sealed pans at 10 °C/min; (**b**) FTIR spectra; (**c**) TG and (**d**) DTG curves at 10 °C/min under nitrogen atmosphere of the corresponding samples.

Complementary thermal and FTIR analyses were conducted to gather information related to the polymerization mechanism of the AMN. Thus, AMNS polymerizations under different environments have been evaluated. Figure 5a shows the non-isothermal DSC thermograms at *β* = 10 °C/min under diverse conditions, and the profiles of these curves are very similar when this thermal polymerization under study is carried out in the presence of an inert gas or air. In addition, the air from an energetic outlook resulted in an average enthalpy polymerization value of 254 kJ/g, which is somewhat less than that estimated under an inert atmosphere. A similar heat of 252 kJ/g was determined when the polymerizations were carried out with sealed pans. This common feature of both polymerizations—air and sealed pan—compared to those conducted under a nitrogen atmosphere can also be seen by molecular spectroscopy through IR analyses, as is shown in Figure 5b. Thus, the FTIR spectra of the polymers based on AMNS synthesized under different environments reflect the structural similarity of the samples obtained with the assistance of an air flow and with sealed pans, but a lower intensity of the peak corresponding to the residual nitrile groups is clearly observed in that sample polymerized under an N_2_ flow, as is illustrated in the inset plot. It is important to note that the air effect is significant from two points of view. On the one hand, the oxygen tolerance of this process can confirm the non-radical nature of the mechanism polymerization. On the other hand, to be able to carry out these syntheses under atmospheric conditions is essential to guaranteeing the optimal selection of polymerization conditions at larger scales in standard laboratories, which can be useful for the potential industrial development of these functional materials.

Interestingly, and instead of the similar enthalpy polymerization values and the FTIR spectra, a clearly different calorimetric behavior is found in the polymerization conducted under sealed pans. This methodology is used to examine the effect of the byproducts generation or volatiles during the course of the thermal reactions. In the present case, accompanying the narrow and well-defined peak that characterizes the AMN polymerization, other peaks of lesser intensity can be observed, which appear at higher temperatures, as is more clearly seen in the inset plot of Figure 5a. In this plot, the deconvolution of the DSC curve by varying the temperature and the intensity of the asymmetric Gaussian peaks is shown. A good fit was found, resulting in three individual narrow peaks plus a fourth broad peak that practically covers the entire temperature range (dashed lines). This result indicates that additional processes can occur under these conditions, and gaseous products released during the thermal AMN polymerization, such as HCN and NH_3_, can also polymerize as side reactions. This result suggests that our system under this condition follows a multistep kinetic, where several reactions (parallel/consecutive or both) simultaneously occur; a certain reaction may dominate at a given temperature, while other reactions may take place at other temperatures. At this point, it is important to take into account that the kinetic modelling of these types of reactions is generally complex, and to kinetically interpret them, some oversimplified methods are available, which address the challenges in this field [37,38]. These results raise the need for additional measurements and complimentary experiments under isothermal conditions to be able to propose a more overwhelming reaction model, which will be the subject of a new study in the near future.

Finally, the TG analyses of the these samples under study, conducted at 10 °C/min and under an inert atmosphere of nitrogen, are now shown in Figure 5c, where it is possible to see their thermal degradation processes, divided into three steps (drying, <225 °C; main decomposition, 225–450 °C; carbonization > 450 °C), which characterized HCN-based polymers [14,15,16,17,36]. Figure 5d displays the corresponding DTG curves, finding the highest intensity peak within the second degradation step at temperatures between 318 and 329 °C, as is observed in the inset plot of this figure. These results denote a thermo-structural resemblance between the samples, especially in those prepared with the presence of air, according to the spectroscopic data discussed above. In addition, they confirm a characteristic of the HCN polymers: their high hydrophilicity, since they can retain adsorbed atmospheric water during polymerization or sample handling, as has been described in the literature [14,36]. These data are further direct evidence that proves that the final product after thermal treatment has a polymeric nature.

**Scheme 1:**
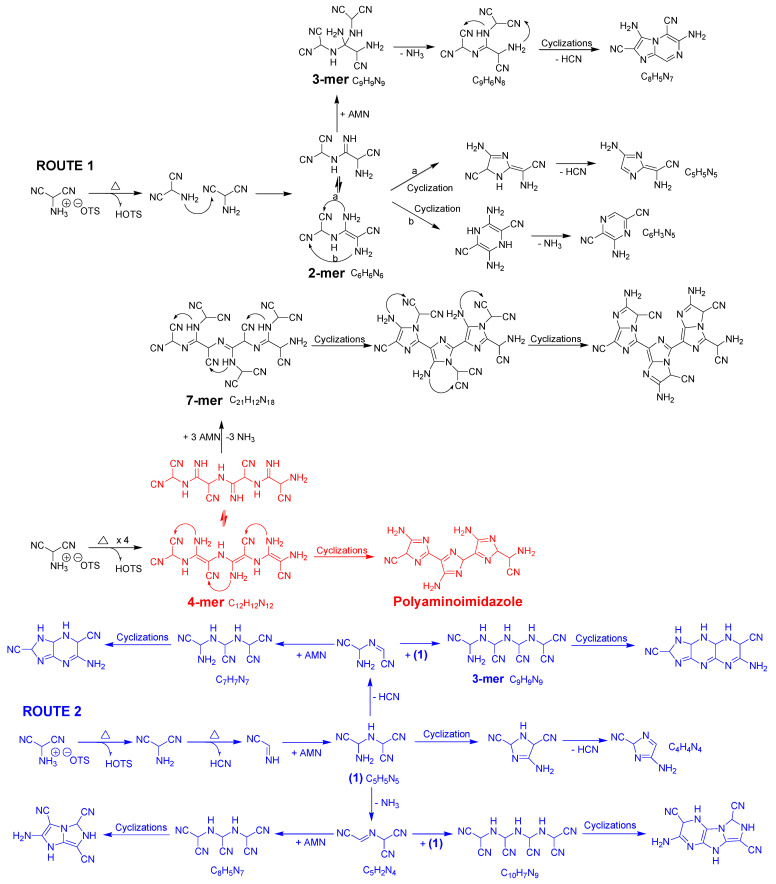
Pathways for the formation of AMN polymers under thermal heating (Δ).

A hypothetical route for AMNS polymerization is described in Scheme 1, taking into account the simultaneous processes of dehydrocyanation and deamination, the FTIR spectroscopic results, and the ruling out of a radical mechanism. Thus, it is possible to distinguish two polymerization routes. The upper part (route 1) shows the step-growth polymerization based on the electrophilicity of the carbon center of the nitrile groups, which makes it susceptible to a variety of nucleophilic addition reactions. Thus, in the dimerization process of the AMNS, after the thermal release of the HOTS, an *N*-unsubstituted imine is formed through an addition reaction between the amine groups of AMN, the nucleophilic agent, and the nitrile groups as the electrophilic core. This step is very illustrative, since it allows us to appreciate the ease of the cyclizations that the formed dimer can undergo to obtain the corresponding aminoimidazole and/or the pyrazole system indicated after the above-mentioned elimination reactions. Logically, the high reactivity of this imine leads to the proposal of an addition reaction of a molecule of AMN to give a trimer, which can evolve new heterocyclic systems with two fused rings. In addition, it is possible to rationalize the polymerization mechanism of the AMN, exemplified in the initial formation of a linear polyenamine 4-mer, and the subsequent cyclization step with the formation of stable five-membered rings (red color). This route leads to polyaminoimidazole, which has been proposed in the literature from the studies of Thissen and Evans regarding the kinetics, chemistry, and morphology of AMN-based polymeric films [8]. However, this synthetic route does not contemplate any of the elimination reactions detected through TG-MS data. From the linear polyenamine or polyamidine, new AMN addition–elimination (NH_3_) reactions yielding, as a final product, a bicyclic system with fused pyrrole and imidazole rings are postulated. At this point, it is relevant to note that this polymerization route is based on the triple bond C≡N, although these polymerization reactions are rarely reported. The cyano group has been polymerized by cationic, anionic, radical, and coordination catalysts; however, the polymerizability of multi-nitrile compounds has scarcely been addressed in the literature [39,40,41].

On the other hand, a second route (blue color) is raised, taking into account the works of L. de Vries about the stability of aminomalonitrile derivaties in a solution [42,43]. In this case, the polymerization is explained through an initial decomposition step of AMN to provide an aminocyanocarbene or its tautomeric iminoacetonitrile (NH=CHCN), with the elimination of HCN. It is important to note that this carbene was detected both during the thermolysis in refluxing toluene and in a basic medium [42,43]. The nucleophilic attack of an AMN molecule on this intermediate species gives the corresponding amine, concretely 2-(amino(cyano)methylamino)malononitrile **(1)**, which can undergo cyclization to generate the imidazole formulated, or new elimination reactions can be proposed. From these new imines, and in the presence of AMN or its derivative **(1)**, it is possible to propose new additions to generate a number of realistic and rational heterocyclic structures constituted by stable five- or six-membered rings with cyano and amine groups through intramolecular cyclizations.

This picture illustrates the complexity of the polymerization mechanism, where different extended conjugated macromolecular systems may also be obtained through the different elimination reactions, possibly with fused rings of imidazole and/or pyrazine or a ladder structure. Further investigation under isothermal conditions, by means of both DSC, which is a suitable technique for the continuous recording of the polymerization course, and preparative scale experiments, are going to test the validity of this kinetic, elucidate mechanistic aspects, and carry out a compressive characterization study. This is mandatory for the development of these new polymeric materials, taking into account that semiconducting polymers-doped HOTS presents relevant sensing, magnetic, and conductive properties [44]. Thus, for example, spongy HOTS-doped polypyrroles with an extraordinary rate performance have been used as durable anodes of sodium-ion batteries [45].

## 4. Conclusions

AMN is a singular molecule that has gained considerable attention among the scientific community in diverse areas, such as heterocyclic organic synthesis, materials science, and, especially, prebiotic chemistry. Herein, an extended DSC examination under non-isothermal conditions from AMNS has revealed the AMN thermally induced polymerization with the proven generation of HCN-derived polymers plus unmodified HOTS. This result encourages further investigation into the nature of these final mixtures obtained by the thermal activation of AMNS, since semiconducting polymers, such as polypyrroles doped with acid, present interesting electrochemical applications. Moreover, the thermoanalytical measurements described here demonstrated that the bulk AMN polymerization is notably easy and reproducible, and it can be considered a very fast reactive system. From these conclusive dynamic DSC data, the first systematic kinetics approach has been described. With the application of differential and integral iso-conversional methods, such as Friedman, FWO, KAS, and Starink, the variation in the apparent *E_α_* at different conversion degrees showed a transition from a kinetic- to a diffusion-controlled regime. The polymerization kinetic was consistent with an apparent self-acceleration model, d*α*/d*t* = *k α^m^* (1 − *α*)*^n^*, with an autocatalytic order *m* ≈ 1 and *n* values dependent on the *β*, and though the kinetic prediction of the reaction rate was in agreement with the experimental values, slight deviations were found. In addition, the thermally initiated polymerizations under sealed pans exhibited a dissimilar behavior. All these results suggest that a complex mechanism involving multiple stages could be considered, and it is necessary to explore the source of this behavior in future studies.

By means of the simultaneous TG-DSC-MS analysis technique under an inert atmosphere, it was possible to gain insight into the nature of the thermochemical reactions present in the thermal polymerization of AMN under dynamic conditions. From these results, one relevant conclusion was drawn: the weight loss observed for this dinitrile monomer is attributed to deamination and dehydrocyanation processes, which took place during its bulk polymerization. Both elimination reactions occurred simultaneously in practically the same temperature range; however, it was difficult to determine if these reactions were the result of thermal decomposition or collateral processes during bulk polymerization. On the basis of these contributions, new polymerization mechanistic proposals are discussed.

The results reported in this study could shed light on the mechanism of polymerization of this reactive molecule derived from HCN, with potential applications in materials science for the development of advanced multifunctional systems, and they can help in understanding the increase in molecular complexity in prebiotic scenarios.

## Figures and Tables

**Table 1:** The thermal properties of AMNS from the DSC thermograms at various *β*s.

*β* (°C/min)	*T_p_* (°C)	*T_i_* (°C)	*T_f_* (°C)	Δ*H* (J/g)
2.5	168.3	157.5	192.0	307
5	174.9	160.2	207.6	300
10	182.0	160.0	216.4	294
15	186.9	167.4	225.9	289
20	190.9	167.1	236.0	279
25	193.4	167.0	237.1	284

**Table 2:** Conversion to reach the maximum reaction rate and kinetic parameters evaluated for the AMNS bulk polymerizations.

*β* (°C/min)	(d*α*/d*t*)·10^3^ (s^−1^)	*α_p_*	*α_M_*	*α_p_^∞^*	*lnA*	*m*	*n*	*n + m*
2.5	9.1	0.399	0.398	0.401	36.81	1.01	1.53	2.54
5	16.8	0.343	0.339	0.346	37.20	1.04	2.03	3.07
10	29.9	0.306	0.298	0.310	37.16	1.05	2.47	3.52
15	41.8	0.299	0.297	0.306	37.30	1.15	2.72	3.87
20	51.5	0.286	0.259	0.289	36.96	0.88	2.51	3.39
25	60.6	0.281	0.279	0.284	37.36	1.13	2.91	4.04

## Data Availability

Not applicable.
